# Machine Learning Methods in Posture-Related Applications in Children up to 12 Years Old: A Systematic Review

**DOI:** 10.3390/bioengineering12121311

**Published:** 2025-11-29

**Authors:** Markel Rico-González, Carlos D. Gómez-Carmona, Ibrahim Ouergui, Luca Paolo Ardigò

**Affiliations:** 1 Department of Didactics of Music, Plastic and Body Expression, University of Basque Country (UPV-EHU), 48940 Leioa, Spain; markel.rico@ehu.eus; 2 BioVetMed & SportSci Research Group, University of Murcia, 30001 Murcia, Spain; 3 Research Group in Training, Physical Activity and Sports Performance (ENFYRED), Department of Music, Plastic and Body Expression, University of Zaragoza, 44003 Teruel, Spain; 4 Research Group in Training Optimization and Sports Performance (GOERD), University of Extremadura, 10071 Caceres, Spain; 5 High Institute of Sport and Physical Education of Kef, University of Jendouba, Kef 7100, Tunisia; brahim.ouerghi@issepkef.u-jendouba.tn; 6 Sport Sciences, Health and Movement, UR22JS01, University of Jendouba, Kef 7100, Tunisia; 7 Department of Teacher Education, NLA University College, 0166 Oslo, Norway

**Keywords:** technology, machine learning, prediction, computer science, health

## Abstract

One of the most important factors in how infants and young children learn to move is postural control. This systematic review aims to evaluate the machine learning methods in posture-related applications for children aged 0–12. Following PRISMA guidelines, we systematically searched the PubMed, Web of Sciences, SCOPUS, and ProQuest Central databases. Twenty-two studies were included in the qualitative synthesis following screening of 199 articles, with methodological quality assessed as moderate to good using the MINORS scale (scores ranging from 8/16 to 19/24). The reviewed research involved diverse samples of infants and children up to 12 years old, employing sensor-based technologies such as inertial measurement units, force plates, pressure mats, and video cameras to extract kinematic and postural features for machine learning applications. Reported accuracies, typically exceeding 85%, reflected considerable methodological heterogeneity related to sensor modality, data quality, and model architecture. Algorithms such as Random Forest, SVM, and CNN were most frequently and effectively applied for posture classification, early detection of developmental delays, and diagnosis of conditions such as cerebral palsy and autism spectrum disorder, demonstrating promising potential for at-home monitoring and clinical interventions.

## 1. Introduction

Postural control is considered one of the critical aspects that designs how newborns and children learn to move, supporting balance, coordination, and interaction with their environment [1]. This can be achieved by the adaptation of different systems such as neuromuscular, sensory, and skeletal ones and their capacity to adapt quickly to both internal growth and external stress across the first months and years of life, inducing rapid changes in body alignment and movement patterns [2,3]. Correct posture in early life is crucial not only for the health of the musculoskeletal system and movement efficiency, but also for the development of motor learning and the independence of functional capacity [4,5]. However, early postural deviations—such as forward head posture, trunk asymmetry, or excessive kyphosis or scoliosis—can lead to compensation in terms of movement patterns, functional limitations, and increased risk of pain or musculoskeletal disorders later in life [6].

Visual observation, observational milestones, goniometric measurement, and photographic or 2D video analysis are commonly used to assess posture in young children in various settings [7,8]. Such methods provide valuable information, but they may be subjective, limited in temporal and spatial sampling, and may require specialized equipment or settings [9]. High-precision laboratory tools such as motion capture systems and force plates can result in accurate, reliable, and rich kinematic and kinetic data, but are resource-intensive and not feasible for routine clinical use or home-based monitoring due to their high cost, specialized personnel requirements, and controlled environment needs, with young children often unable to adapt well to laboratory settings [10,11]. This gap between the need for early, frequent, and ecologically valid assessment and the limitations of current methods has spurred interest in more scalable, objective, and technology-driven alternatives [9,12].

Recent advancements in machine learning (ML)—a subset of artificial intelligence (AI) that empowers computers to identify, categorize, and predict complex patterns in data— have revolutionized methodologies for posture and movement analysis [13,14,15]. ML models use finite details, learn how things are related in a non-linear manner, and make predictions from massive amounts of sensor, video, or image data sets [13,15,16]. While many existing ML studies have relied on laboratory-based technologies—such as wearable inertial measurement units (IMUs) to track trunk and limb movements, pressure mats, and force plates to assess postural load distribution [15,16,17,18], or 2D/depth-camera-based systems to extract keypoints and joint angles [19]—these tools mainly serve as research-grade references and remain impractical for widespread pediatric screening or routine follow-up. However, recent research showed that technological progress has enabled ML models to analyze infant postural control using accessible data sources like standard video recordings and accelerometers [1,17]. Studies have utilized computer algorithms to assess posture from images on glass platforms and evaluated infant movements through video data [1,18]. The combination of accelerometers and video has also proven its effectiveness in quantifying postural alignment in children who cannot sit independently [17]. These innovations demonstrate the efficacy of using low-cost data sources for advanced infant postural assessment in clinical and research environments. Nonetheless, this body of work demonstrates the analytic potential of ML-based posture evaluation in children, using classifiers such as Support Vector Machines (SVMs), Random Forests (RFs), and deep learning architectures like Convolutional Neural Networks (CNNs) [13,19].

Despite these advances, research specifically applying ML to pediatric posture assessment remains limited and methodologically diverse [13]. Children grow rapidly and differently from adults, with different motor patterns and unique sensor/algorithm requirements [9,20]. Additional challenges are the ethical issues (including privacy and consent), small datasets for pediatrics, and differences in how measurements are taken [9,20]. While previous reviews have addressed posture detection or ML in general, a focused synthesis of pediatric posture applications using machine learning is lacking [9,16].

Thus, this systematic review aimed to comprehensively evaluate the ML methods in posture-related applications for children aged 0–12. The specific aims were to (1) catalog sensor and vision-based technologies for pediatric posture monitoring; (2) examine ML algorithms used and performance classification; (3) clinical applications and diagnostic capabilities; (4) describe naturalistic assessment and home-based monitoring; and (5) identify methodological gaps, ethical issues, and future research directions.

## 2. Materials and Methods

### 2.1. Experimental Approach to the Problem

This systematic review followed the Preferred Reporting Items for Systematic Reviews and Meta-Analyses (PRISMA) guidelines [21] and adhered to established guidelines for performing systematic reviews in sport sciences [22]. The review protocol was developed to ensure comprehensive coverage of relevant literature while maintaining methodological rigor. The systematic review was registered (PROSPERO: CRD420251218895).

### 2.2. Information Sources

A comprehensive search was conducted across four databases: PubMed, Web of Sciences, SCOPUS, and ProQuest Central. The search encompassed all published literature prior to 29 August 2025. This combination of databases was selected to ensure broad coverage of both medical and sports science literature.

### 2.3. Search Strategy

The PICO (Patient, Problem, or Population–Intervention or Exposure–Comparison, Control, or Comparator–Outcome[s]) framework was implemented to structure the search strategy and ensure systematic coverage of relevant literature [21]. To maintain transparency, the authors were not blinded to journal names or manuscript authors. Language filters were applied to include English and Spanish publications only. The search terms were carefully selected to capture all relevant literature on information technologies and machine learning in primary school settings. The final search string was: (child* OR infant*) AND (“machine learning”) AND (postur*).

### 2.4. Eligibility Criteria

The authors introduced the search string into databases and downloaded the title, author names, journal, and date of all the articles that appeared in the search. Once the Excel spreadsheet was organized, all duplicates were removed, and the remaining articles were evaluated for their eligibility (Table 1). If the authors found articles that had not appeared in the search, they included them in the Excel document as “included from external sources”.

### 2.5. Data Extraction

A standardized data extraction process was implemented using an Excel spreadsheet developed in accordance with the Cochrane Consumers and Communication Review Group’s data extraction template [23]. The spreadsheet facilitated systematic assessment of inclusion and exclusion requirements for all selected studies. Two authors independently conducted the extraction process, with any disagreements resolved through discussion until consensus was reached. Full documentation was maintained for excluded articles, including specific reasons for exclusion. All data were systematically recorded and stored in the spreadsheet.

### 2.6. Assessment of Study Methodology

The methodological quality was assessed using the methodological index for non-randomized studies (MINORS) [24]. The MINORS scale is a list that contains 8 essential points, and it is expanded to 12 points when the studies to be treated are comparative. In this case, it was assessed considering 9 items (out of 18 points) due to the non-possibility of applying (NA) three of them. The score that each section receives can be from 0 to 2, depending on the quality obtained by each point (0 = Low quality; 1 = Medium quality; 2 = Good quality).

## 3. Results

### 3.1. Identification and Selection of Studies

**Figure 1:**
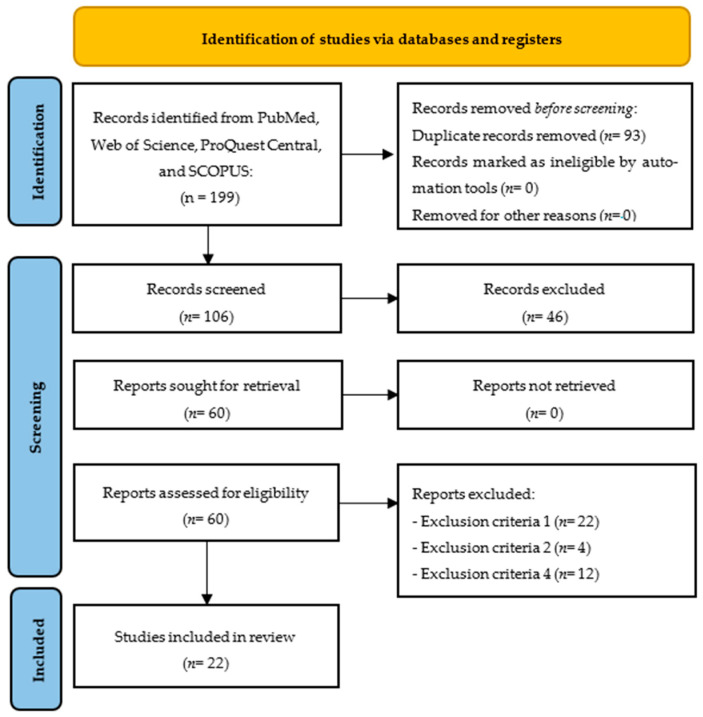
Flow diagram of the study.

After analyzing all databases (PubMed: 27; Web of Science: 78; ProQuest Central: 14; SCOPUS: 80), the contents of 199 articles were checked; we detected, at the initial stage, 93 duplicate articles. Then, the authors analyzed whether each of the remaining 106 articles met all inclusion criteria, resulting in the elimination of 22 articles by exclusion criterion number one, exclusion criterion number two (n = 4), exclusion criterion number four (n = 12), and exclusion criterion number six (n = 46). The remaining 22 articles were included in the qualitative synthesis of the systematic review (Figure 1).

### 3.2. Quality Assessment

The methodological quality of the 21 included studies was assessed using the MINORS scale, which evaluates up to 12 items (scored 0–2 each, with some non-applicable), resulting in total scores ranging from 8/16 to 19/24 (Table 2). Most studies scored good quality (2/2) on clearly defined objectives (item 1), assessments adjusted to objectives (item 4), neutral evaluations (item 5, where applicable), and appropriate statistical analysis (item 12), but consistently low quality (0/2) on prospective data collection (item 3) and sample size estimation (item 8). Overall, the studies exhibited medium to good quality, with average scores around 13/18 for non-comparative designs, indicating robust objectives and analyses but room for improvement in prospective planning and follow-up consistency.

### 3.3. Study Characteristics

#### 3.3.1. Sample

The included studies encompassed diverse pediatric populations, primarily infants and children up to 12 years old, with sample sizes ranging from small cohorts (e.g., 10 children in Kim et al. [13] and Eken et al. [33]) to larger groups (e.g., 514 primary school children in Tao et al. [34]). Participants included typically developing infants and children, as well as those with neurodevelopmental conditions such as autism spectrum disorder (ASD; n = 50 in Li et al. [15]), cerebral palsy (CP; up to 140 across Khaksar et al. [16] and Bertoncelli et al. [29]), HIV encephalopathy (n = 10 in Eken et al. [33]), and forward head posture (FHP; n = 514 in Tao et al. [34]). Some studies incorporated simulation models (e.g., child occupant model in Li et al. [31]) or synthetic data (e.g., MINI-RGBD dataset in Gama et al. [37]), while others focused on at-risk or delayed development groups, ensuring representation of both healthy and clinical samples.

#### 3.3.2. Data Collection Methods

Data collection predominantly utilized sensor-based and imaging technologies, including inertial measurement units (IMUs) with accelerometers and gyroscopes (e.g., at 50–100 Hz in Airaksinen et al. [25,36], Franchak et al. [32,38], and Duda-Goławska et al. [40]), force plates for center of pressure (COP) analysis (e.g., 60–200 Hz in Li et al. [15] and Arias Valdivia et al. [27]), and video cameras for pose estimation (e.g., 15–60 fps in Yang et al. [26], Gama et al. [37], and Rachwani et al. [41]). Other methods involved pressure sensor mats for sitting posture (e.g., 8 × 8 grids at 10 Hz in Kim et al. [13,14] and Lee et al. [28]), Kinect depth cameras for anthropometric measurements (in Tao et al. [34]), and custom wearables like smart jumpsuits or suits (in Airaksinen et al. [25,36]). The features extracted included kinematic variables (e.g., joint angles, velocities), pressure distributions, and time-series data from short trials (e.g., 20–30 s) or full-day recordings, often in controlled or naturalistic environments.

#### 3.3.3. Study Settings and Research Focus

Studies were conducted in varied settings, including laboratories for controlled assessments (e.g., biomechanics labs in Li et al. [15], Arias Valdivia et al. [27], and Eken et al. [33]), home environments for naturalistic data (e.g., video recordings in Yang et al. [26], Franchak et al. [32,38], and Ledwon et al. [35]), clinical hospitals (e.g., for CP patients in Bertoncelli et al. [29] and Khaksar et al. [16]), and schools (e.g., for FHP screening in Tao et al. [34]). The primary research focus was on posture-related applications, such as classifying gross motor milestones and developmental delays in infants (e.g., Airaksinen et al. [4,20] and Yang et al. [6]), identifying sitting postures to prevent musculoskeletal issues (e.g., Kim et al. [13,14] and Lee et al. [28]), diagnosing conditions like ASD, CP, or FHP via postural sway or movement patterns (e.g., Li et al. [15], Arias Valdivia et al. [27], and Tao et al. [34]), and predicting injury risks or asymmetries (e.g., Li et al. [31] and Ledwon et al. [35]). Emphasis was placed on early detection, automated screening, and longitudinal tracking for clinical and rehabilitative purposes.

#### 3.3.4. Machine Learning Implementation

Machine learning implementations featured a range of algorithms, with classifiers like Random Forest (e.g., achieving 94% accuracy in Yang et al. [26] and 87.75–89.39% in Khaksar et al. [16]), Support Vector Machines (SVMs; e.g., 88–97.37% accuracy in Sukhadia & Kamboj [30] and Ali & Mohamed [39]), and Convolutional Neural Networks (CNNs; e.g., 95.3–99.66% for posture classification in Kim et al. [13], Kim et al. [14], Lee et al. [28], and Airaksinen et al. [36]) being prominent. Deep learning models such as LSTM/GRU variants (e.g., 76.43% accuracy in Arias Valdivia et al. [27]) and pose estimation networks (e.g., ViTPose with 92% OKS in Gama et al. [37]) were used for temporal and video data, often with cross-validation yielding high metrics (e.g., AUC 0.865–0.98, F1-scores 0.617–0.955). Key applications included real-time posture monitoring, early disorder prediction, and injury risk assessment, with conclusions highlighting feasibility for at-home tools, clinical diagnostics, and personalized interventions, though performance varied with user familiarity and data quality. Although Table 3 reports accuracy ranges for various algorithms, direct comparison across studies is limited due to the absence of standardized evaluation metrics and heterogeneous methodologies. Differences in reported accuracies may stem from variations in sensor modalities (e.g., IMU, pressure mat, camera), data quality, preprocessing pipelines, and model architectures rather than intrinsic algorithmic superiority. Future research should adopt consistent benchmarking protocols and unified performance indicators to enable fairer cross-study comparisons of ML approaches in pediatric posture analysis.

To facilitate cross-study comparison, Table 4 summarizes the main machine learning models applied across the reviewed studies, detailing their corresponding tasks, sensing modalities, dataset characteristics, key performance indicators, and, where available, inference times. This comparative overview highlights the methodological diversity underlying reported accuracies and underscores the influence of factors such as sensor type, data volume, and computational approach on model performance.

## 4. Discussion

Traditional pediatric posture assessment methods are subjective, intermittent, and clinic-based [7,8,9], while machine learning applications in pediatric contexts remain understudied despite rapid advances in adult populations. Critical gaps exist regarding which technologies and algorithms are most effective for children, whether automated systems achieve clinically meaningful accuracy for early detection, and how to enable continuous naturalistic monitoring. This gap is concerning, given that early identification of postural deviations and motor delays enables timely intervention to prevent long-term complications and improve trajectories for children with neurodevelopmental conditions [4,5,6]. Therefore, this systematic review examined the application of machine learning algorithms for posture-related assessments in infants and children up to 12 years old, following PRISMA guidelines [21] and established systematic review protocols [22,23]. The analysis of 22 studies revealed that sensor-based technologies combined with machine learning classifiers achieve very good to excellent accuracy (typically exceeding 85%, range: 76–99%) [42] in detecting developmental delays, classifying postures, and diagnosing neurodevelopmental conditions such as cerebral palsy and autism spectrum disorder [13,14,15,16,25,26,27,28,29,30,31,32,33,34,35,36,37,38,39,40,41]. The predominant technologies employed included inertial measurement units, force plates, pressure sensor mats, and video cameras, with algorithms such as Random Forest, Support Vector Machines, and Convolutional Neural Networks demonstrating superior performance across diverse applications [13,14,15,16,25,26,27,28,29,30,31,32,33,34,35,36,37,38,39,40,41]. These findings highlight the feasibility of automated, objective posture assessment systems for clinical diagnostics, early intervention, and continuous at-home monitoring.

### 4.1. Sensor Technologies and Data Collection Methods

The reviewed studies predominantly utilized three categories of sensing technologies: wearable inertial measurement units, pressure-based systems, and vision-based approaches. Wearable IMUs equipped with accelerometers and gyroscopes emerged as versatile tools, enabling longitudinal tracking of infant motor development in naturalistic home environments [25,33,37,39,41]. These devices demonstrated particular effectiveness in detecting gross motor milestones, with studies reporting detection accuracies ranging from 90.9% to 96.8% for posture classification [25]. The portability and non-invasive nature of IMU-based wearables facilitate extended monitoring periods, addressing a critical limitation of traditional clinical assessments. Validation studies have established that commercially available IMUs provide accurate measurements for infant motor assessment, with accelerometers serving as reliable tools when combined with pressure mattresses for comprehensive kinematic evaluation [43]. Recent advances in wearable sensor networks have demonstrated the feasibility of quantifying full-body movement behaviors in vulnerable populations [44,45]. Furthermore, the integration of multiple sensor locations (typically on limbs and trunk) enabled kinematic analysis of gross body movements and postural orientation that successfully classified five distinct body positions with accuracy [32,36,38]. However, it is important to note that IMU-based systems capture global movement patterns and body segment orientations rather than detailed 3D joint kinematics, which remain more accurately measured by marker-based motion capture systems [43,44].

Pressure sensor arrays and force plates offered complementary advantages for sitting posture analysis and postural control assessment. Studies utilizing film-type force-sensing resistor mats demonstrated that two-dimensional pressure distribution patterns could effectively discriminate between multiple sitting postures [13,14,28]. The conversion of raw pressure data into heat map images proved particularly suitable for Convolutional Neural Network processing, with Kim et al. [14] achieving 97.5% accuracy in classifying seven distinct sitting postures. Force plate systems provided precise quantification of center of pressure dynamics, enabling automated identification of autism spectrum disorder with 90% accuracy through analysis of postural sway characteristics [15]. Vision-based approaches, including standard video cameras and depth sensors, offered the advantage of completely contactless measurement, with pose estimation algorithms successfully extracting gross kinematic features [26,34,36,38,40,43]. Computer vision-based posture analysis systems have emerged as efficient screening tools in clinical practice, demonstrating conformity with radiographic parameters for spinal deformity detection [46].

### 4.2. Machine Learning Algorithms and Classification Performance

Random Forest and Support Vector Machine classifiers demonstrated robust performance across multiple studies. Random Forest achieved notable success in developmental delay classification (94% accuracy) [26] and cerebral palsy detection (87.75–89.39% accuracy) [16], likely attributable to the algorithm’s ability to handle high-dimensional feature spaces and non-linear relationships. The interpretability of decision tree-based models represents an additional advantage in clinical contexts, where understanding feature importance can provide mechanistic insights into postural control deficits and support clinical decision-making [29,30]. Support Vector Machines exhibited comparable efficacy, with reported accuracies ranging from 88% to 97.37% for cerebral palsy prediction [31,40], demonstrating particular effectiveness when combined with carefully engineered frequency-domain features derived from inertial sensor data [16].

Convolutional Neural Networks emerged as the preferred architecture for image-based posture classification tasks. Multiple studies reported CNN accuracies exceeding 95% for sitting posture classification from pressure sensor arrays [13,14,28], with Lee et al. [28] achieving 99.66% accuracy for user-specific models. The superior performance of CNNs compared to traditional machine learning algorithms (Artificial Neural Networks and Multi-layer Neural Networks achieved 82.9% and 88.7%, respectively) [14] underscores the value of deep learning architectures for pattern recognition in high-dimensional sensor data. Recurrent neural network variants, including Long-Short-Term Memory (i.e., Long Short-Term Memory is an improvement over standard RNN, designed to address the difficulty of learning long-term dependencies) and Gated Recurrent Units, showed promise for temporal sequence modeling of postural sway dynamics [27]. Recent pose estimation networks, particularly vision transformer-based approaches, demonstrated state-of-the-art performance (92% Object Keypoint Similarity) for automated infant pose tracking from video [36], suggesting that transfer learning from large-scale human pose datasets can effectively address the challenges of limited pediatric training data. Comparative evaluations of deep neural network pose estimation methods (including AlphaPose, DeepLabCut, Detectron2, HRNet, MediaPipe, OpenPose, and ViTPose) on infant videos revealed that modern architectures achieve competitive performance without additional fine-tuning, with ViTPose demonstrating superior accuracy across multiple benchmarking metrics [37,46]. Analysis of end-to-end neural network architectures for infant motility assessment has demonstrated that optimization of encoder modules and data augmentation strategies can significantly enhance classifier robustness, particularly under noisy conditions in real-world scenarios [36,47].

### 4.3. Clinical Applications and Diagnostic Capabilities

The reviewed studies demonstrate substantial potential for machine learning-assisted early detection of neurodevelopmental disorders through posture and movement analysis. Automated identification of autism spectrum disorder from postural control patterns represents a particularly promising application, with Li et al. [15] reporting 100% specificity and 90% overall accuracy using center of pressure complexity measures. The good specificity suggests potential utility as a screening tool to rule out ASD, though the moderate sensitivity (82.6%) indicates that confirmatory assessment would remain necessary for suspected cases. Cerebral palsy detection and subtype classification emerged as another well-established application domain, with multiple studies achieving accuracy levels considered clinically meaningful for screening applications (>80% accuracy, with sensitivity and specificity adequate to reduce false negatives/positives compared to standard clinical assessment) [16,27,29,30,39]. Arias Valdivia et al. [27] successfully differentiated hemiplegia from diplegia with 76.43% accuracy using postural sway time-series data, while Khaksar et al. [16] distinguished children with cerebral palsy from typically developing controls with 89.39% accuracy in toddlers using wrist movement patterns during functional tasks.

The progression from diagnostic classification to continuous monitoring and quantitative assessment of motor development represents an emerging frontier with significant clinical implications. Airaksinen et al. [25] demonstrated that automated gross motor milestone detection could achieve performance comparable to expert clinician agreement (correlation of 0.93 between automated scores and standardized assessments), enabling longitudinal tracking of motor trajectory without repeated clinical visits. This capability addresses critical gaps in current pediatric care, where developmental surveillance often relies on infrequent clinic visits and subjective parental report. Validation studies have confirmed that quantified assessment of infant motor performance using fully automated analysis pipelines can replicate across independent cohorts from out-of-hospital recordings [25,36]. The extension of postural monitoring to preventive applications, including forward head posture screening in school populations [18] and real-time sitting posture correction systems [13,14,28], illustrates the broader applicability of these technologies beyond clinical diagnosis. The Random Forest model developed by Tao et al. [34] identified body mass index and sedentary behavior patterns as key risk factors for forward head posture (AUC = 0.865), facilitating targeted interventions for at-risk children. However, the transition from controlled research settings to clinical implementation will require validation across diverse populations, standardization of assessment protocols, and integration with existing clinical workflows to ensure that algorithmic predictions appropriately complement rather than replace clinical expertise [48,49].

### 4.4. Naturalistic Assessment and Home-Based Monitoring

The capability to conduct posture and movement assessment in naturalistic home environments represents a paradigm shift from traditional laboratory-based evaluations. Multiple studies successfully demonstrated that wearable sensors embedded in everyday garments (smart jumpsuits, leggings) could capture motor behavior representative of real-world activities across entire days of infant activity [25,32,36,38]. Franchak et al. [32] reported that distal sensor placement (ankles and hips) achieved 86% accuracy for body position classification during naturalistic play, while proximal placement improved accuracy to 97.9%, establishing optimal sensor configurations for at-home monitoring. The feasibility of full-day recording addresses a fundamental limitation of clinical assessments, which typically capture only brief trials that may not represent typical motor patterns.

Vision-based approaches offer the advantage of leveraging video recordings captured by parents using standard smartphones. Yang et al. [26] achieved 94% accuracy in identifying gross motor developmental delays from home videos using pose estimation combined with Random Forest classification, demonstrating that accessible technology can enable remote screening in resource-limited settings. Gama et al. [37] revealed that modern architectures (ViTPose, HRNet) achieve good pose estimation accuracy (Object Keypoint Similarity of 0.92) without requiring infant-specific training data, facilitating immediate deployment for large-scale screening initiatives. Recent analyses confirmed that pose estimation methods provide accurate tracking of gross body movements and postural configurations using low-cost imaging systems with processing speeds suitable for real-time applications, though these methods capture global movement patterns rather than detailed 3D joint kinematics [37,46]. Rachwani et al. [41] demonstrated that video-based kinematic analysis could quantify trunk stability and movement coordination in naturalistic sitting contexts, while Ledwoń et al. [35] achieved 92.03% accuracy and 93.26% sensitivity for automated postural asymmetry assessment. The convergence of wearable and vision-based approaches toward unobtrusive, longitudinal assessment creates opportunities for early identification of subtle developmental deviations that may not manifest during brief clinical examinations.

### 4.5. Limitations and Practical Applications

Several methodological limitations warrant consideration when interpreting the findings of this systematic review. The MINORS quality assessment revealed consistent weaknesses in prospective data collection (most studies scored 0/2 on item 3) and sample size estimation (item 8). The predominance of cross-sectional designs limits the capacity to establish predictive validity and determine whether automated assessments can reliably forecast long-term developmental outcomes, a critical requirement for clinical decision support systems. Sample sizes ranged considerably from small pilot studies with 10 participants to larger cohorts of 514 children. Many studies lacked external validation on independent datasets, raising concerns about generalizability across diverse populations, cultural contexts, and clinical settings. The performance degradation observed when machine learning models encountered unfamiliar users underscores the importance of developing person-independent classification approaches or implementing user calibration procedures. Furthermore, the heterogeneity in sensor specifications, data collection protocols, feature extraction methods, and machine learning implementations across studies precludes direct comparison of algorithmic performance and complicates the identification of optimal methodological approaches. These methodological concerns align with broader challenges in clinical AI validation, where longitudinal evaluation and standardized interpretability metrics remain essential for responsible implementation.

A critical limitation across the reviewed studies is the limited implementation and reporting of explainable artificial intelligence (XAI) methods. Among the 22 included studies, only one explicitly employed XAI techniques (Slijepcevic et al. [19]), which applied explainability methods to gait analysis in children with cerebral palsy to identify clinically relevant biomechanical patterns. Several studies using tree-based algorithms (Random Forest [16,26,27] and Support Vector Machines [30,39]) provided inherent interpretability through feature importance rankings, which identified key predictors such as body mass and sedentary behavior patterns [34]. However, most studies employing deep learning architectures—including Convolutional Neural Networks [13,14,28,36], recurrent neural networks [27], and vision transformers [37]—achieved good accuracy (>95%) without reporting explainability analyses such as GradCAM, attention visualization, or SHAP values. This lack of transparency presents a significant barrier to clinical adoption, as clinicians require an understanding of model reasoning to verify clinical relevance, identify potential biases, and maintain appropriate oversight of algorithmic recommendations [48,49]. Future research should prioritize implementing and reporting XAI methods appropriate to each algorithm type: GradCAM and saliency visualization for CNNs, attention mechanisms for temporal models, and SHAP or LIME for model-agnostic interpretability [19,48,49].

Despite these limitations, the reviewed evidence establishes clear practical applications for machine learning-assisted posture assessment in pediatric healthcare and educational settings. Smart chair systems for real-time sitting posture monitoring and wearable-based motor milestone tracking demonstrate very good to excellent accuracy for deployment in school and primary care settings. Integration with routine screening could enhance the efficiency of health programs, while continuous at-home monitoring provides objective metrics of therapy response for children with neurodevelopmental conditions. Video-based screening tools hold particular promise for expanding developmental surveillance to underserved populations. Successful clinical translation requires explainable artificial intelligence approaches that ensure transparency and interpretability of model decisions, addressing the “black box” problem that limits clinical trust and adoption. The development of clinical decision support systems incorporating explainability methods such as SHAP and LIME can enhance clinician understanding of algorithmic recommendations while maintaining diagnostic accuracy. Collaboration among researchers, clinicians, and regulatory bodies remains essential to establish validation standards, ensure data privacy protections, and develop implementation frameworks that preserve clinical judgment while leveraging the quantitative capabilities of machine learning-assisted assessment.

Future research should prioritize several critical directions. First, ML-driven adaptive interventions could transform pediatric posture management from static assessment to dynamic, personalized treatment systems. Smart chair systems [13,14,28] could evolve to provide adaptive real-time feedback based on individual response patterns, while wearable systems [25,36] could integrate with mobile applications to deliver personalized motor development activities with difficulty automatically adjusted based on progress trajectories. Such adaptive systems employing reinforcement learning and continuous monitoring could optimize intervention timing and content for maximum effectiveness while improving treatment adherence. Second, longitudinal prospective studies are needed to establish predictive validity for long-term developmental outcomes, as current cross-sectional designs cannot determine whether automated assessments provide meaningful prognostic information. Third, external validation across diverse populations is essential to establish generalizability and identify algorithmic biases. Fourth, standardization of protocols, feature extraction methods, and benchmark datasets would facilitate meaningful comparisons and accelerate methodological innovation. Finally, implementation science studies examining clinical adoption barriers, training requirements, and workflow integration are needed to translate research prototypes into deployable clinical tools.

## 5. Conclusions

Machine learning algorithms combined with sensor-based and vision-based technologies demonstrate substantial promise for objective, automated assessment of posture and motor development in infants and children up to 12 years old, with studies consistently achieving accuracy rates exceeding 85%, with very good to excellent accuracy (range: 76–99%) for detecting developmental delays, classifying postures, and diagnosing neurodevelopmental conditions such as autism spectrum disorder and cerebral palsy. Key technologies, including inertial measurement units, pressure sensor arrays, force plates, and video cameras, paired with algorithms such as Random Forest, Support Vector Machines, and Convolutional Neural Networks, enable diverse applications ranging from early disorder detection and real-time sitting posture monitoring to longitudinal tracking of motor milestones in naturalistic home environments.

However, critical gaps exist: only 1 of 22 studies explicitly implemented XAI methods; most employed cross-sectional designs limiting predictive validity; and external validation across diverse populations remains insufficient. Future priorities include developing ML-driven adaptive interventions for personalized treatment optimization, conducting longitudinal studies, implementing explainable AI approaches, and performing implementation science research. Successful clinical translation requires addressing the “black box” problem through explainable artificial intelligence approaches, standardizing assessment protocols across studies, validating models on independent datasets, and implementing person-independent classification strategies to ensure that algorithmic tools appropriately complement rather than replace clinical expertise while expanding developmental surveillance to underserved populations.

## Figures and Tables

**Table 1:** Inclusion and exclusion criteria.

Item	Inclusion	Exclusion
Population	Children as participants (until 12 years old)	Studies with non-child participants (more than 12 years old)
Intervention or Exposure	Studies that used machine learning	Studies that did not use machine learning
Comparation	Not applicable	Not applicable
Outcome[s]	Any result (validity or reliability studies, predictions…) related to children’s body posture	Any result not related to children’s body posture
Other criteria	Peer-reviewed full-text studies published in original journal articles	Non-peer-reviewed journal articles Non-original full-text studies (conference papers…) Reviews

**Table 2:** Methodological assessment of the included studies.

Reference	1	2	3	4	5	6	7	8	9	10	11	12	Score
Airaksinen et al. [25]	2	1	0	2	2	2	2	1	-	-	-	2	14/18
Li et al. [15]	2	1	0	2	2	2	2	2	-	-	-	2	15/18
Yang et al. [26]	2	1	1	2	2	-	-	0	-	-	-	2	10/14
Kim et al. [13]	2	1	0	2	2	2	2	0	-	-	-	2	13/18
Arias Valdivia et al. [27]	2	1	0	2	2	-	-	0	-	-	-	2	9/14
Khaksar et al. [16]	2	1	1	2	1	2	2	1	2	2	1	2	19/24
Kim et al. [14]	2	1	0	2	2	2	2	0	-	-	-	2	13/18
Lee et al. [28]	2	1	0	2	2	2	2	0	-	-	-	2	13/18
Bertoncelli et al. [29]	2	1	2	2	2	2	2	0	-	-	-	2	15/18
Sukhadia & Kamboj [30]	2	1	0	2	1	-	0	0	-	-	-	2	8/16
Li et al. [31]	2	1	0	2	2	2	-	0	-	-	-	2	11/16
Franchak et al. [32]	2	1	0	2	2	2	2	0	-	-	-	2	13/18
Eken et al. [33]	2	1	0	2	2	2	2	0	-	-	-	2	13/18
Tao et al. [34]	2	1	0	2	2	0	0	0	-	-	-	2	9/18
Ledwon et al. [35]	2	1	0	2	2	2	2	0	-	-	-	2	11/18
Airaksinen et al. [36]	2	1	0	2	2	2	2	0	-	-	-	2	13/18
Gama et al. [37]	2	1	0	2	2	-	-	0	-	-	-	2	9/14
Franchak et al. [38]	2	1	0	2	2	2	2	0	-	-	-	2	13/18
Ali & Mohamed [39]	2	0	0	2	2	2	-	0	-	-	-	2	10/16
Duda-Goławska et al. [40]	2	1	0	2	2	2	1	0	-	-	-	2	13/18
Rachwani et al. [41]	2	1	0	2	2	2	2	0	-	-	-	2	13/18

Note: The MINORS checklist: clearly defined objective (item 1); inclusion of patients consecutively (item 2); information collected retrospectively (item 3); assessments adjusted to objective (item 4); evaluations carried out in a neutral way (item 5); follow-up phase consistent with the objective (item 6); dropout rate during follow-up less than 5% (item 7); prospective estimation of sample size (item 8); adequate control group (item 9); simultaneous groups (item 10); homogeneous starting groups (item 11); and appropriate statistical analysis (item 12).

**Table 3:** Main characteristics and findings of machine learning applications.

Ref.	Participants’ Characteristics	Activity Registration				Aim of Prediction Related to Posture	MLe/DL Accuracy		Conclusions	Practical Application for Predicting
		Tool	Tool’s Specifications	Location	Attributes/Features/Variables		Algorithm	%		
Airaksinen et al. [25]	134 infants (4–22 months); Cohort 1 (n = 97, typically developing), Cohort 2 (n = 37, developmental risk)	MAIJU wearable suit (multi-sensor)	Accelerometer and gyroscope; 52 Hz (1024 samples/s); Bluetooth to mobile device	Limbs (standard locations on each limb)	Second-by-second posture and movement detections (“MAIJU features”): supine, prone, sitting, crawling, standing, walking, etc.	To detect gross motor milestones (GMMs), quantify time spent in key postures, and track holistic motor development (BIMS score)	Support Vector Machine (SVM) for GMM detection; Linear Mixed-Effects Model (LME) for BIMS	GMM detection accuracy: 90.9–96.8% (cross-val. and external val.); BIMS vs. age correlation (Spearman’s ρ): 0.93	Unsupervised at-home wearable measurements can accurately and automatically quantify infant gross motor skills, with performance comparable to expert agreement levels.	Objective, at-home assessment of infant motor development for early detection of delays, clinical support, and longitudinal tracking in healthcare and research.
Li et al. [15]	50 children (25 ASD, 25 TD); age 5–12 years; mild ASD (level 1)	Force plate (Kistler Instrument Corp., Winterthur, Switzerland)	60 Hz sampling rate; 20 s quiet standing trials; eyes open and eyes closed conditions	Biomechanics laboratory	COP linear displacements (AP, ML), total distance, sway area, sample entropy (complexity)—12 features total (6 variables × 2 conditions)	Automated identification of ASD based on postural control patterns	Naïve Bayes (best performer among 6 classifiers)	Accuracy: 0.900 Sensitivity: 0.826 Specificity: 1.000 Precision: 1.000 F1 Score: 0.898	Naïve Bayes best identified ASD postural control with high accuracy and specificity. COP complexity improved classification by ~4%.	Potential as an early diagnostic tool for ASD using postural sway biomarkers; supports computer-aided diagnosis with minimal human intervention.
Yang et al. [26]	90 infants (aged 2–6 months); 26 with developmental delays, 64 typically developing	Video camera (home recordings)	15 frames per second	Home environment	227 features extracted (106 significant after ANOVA): • Kinematic (speed, acceleration of elbows, wrists, knees, ankles) • Joint angles (shoulders, elbows, hips, knees) • Angular velocity and acceleration • Entropy of movement	Automatic classification of gross motor development (normal vs. abnormal)	Random Forest	Accuracy: 94% F1-score: 0.94 AUC: 0.98	The ViTPose model provided the best pose recognition. A Random Forest classifier using 106 significant features achieved high performance in automatically identifying infants with gross motor delays from home videos.	Enables remote, automated early screening for gross motor developmental delays in infants using simple home videos, facilitating timely intervention, especially in resource-limited areas.
Kim et al. [13]	10 children; age not specified (school-aged); no health conditions reported	Sensing cushion with pressure sensor mat	Film-type FSR sensor (8 × 8 grid); 318 × 318 mm area; 12-bit data; Bluetooth transfer	Seat cushion of a child’s chair	Two-dimensional pressure distribution images (8 × 8 pixels); raw pressure values used as input features.	To classify children’s sitting postures into five categories in real time	CNN (LeNet-5 modified), NB, DT, NN, MLR, SVM	CNN: 95.3% (Avg. accuracy, individual validation); NB: 87.1%; MLR: 84.5%; DT: 79.4%; NN: 92.1%; SVM: 94.2%	The CNN algorithm outperformed conventional machine learning algorithms for classifying sitting postures from pressure distribution data. Accuracy was influenced by the child’s body weight.	Enables the development of a smart chair or real-time posture monitoring system to promote correct sitting habits and prevent musculoskeletal disorders in children.
Arias Valdivia et al. [27]	57 pediatric patients (7–14 years, 9.2 ± 1.8 years; 29 males, 28 females) diagnosed with hemiplegia (n = 35) or diplegia (n = 22)	AMTI force platform	Force plate; 200 Hz sampling rate; measures forces (F_x_, F_y_, F_z_) and moments (M_x_, M_y_ M_z_)	Laboratory setting; participants stood barefoot on the platform	Center of pressure (COP) coordinates, velocity (VEL_x_, VEL_y_), standard deviation (STDx, STDy), area of ellipse; time series data derived from 30 s trials.	To classify type of cerebral palsy (hemiplegia vs. diplegia) based on postural control analysis during quiet standing	LSTM, GRU, BiLSTM, BiGRU, ARIMA (BiGRU performed best)	Accuracy: 76.43% (BiGRU)	BiGRU model most effectively captured temporal dependencies in postural sway for classification, outperforming traditional methods and unidirectional models; offers a non-invasive, objective diagnostic aid.	Provides a clinical decision-support tool for differentiating CP subtypes using brief, static postural control tests, aiding in early and accurate diagnosis and personalized intervention planning.
Khaksar et al. [16]	Two age groups: • ~15 years (MIT trial): 89 with CP, 30 without CP • ~3 years (iWHOT trial): 51 with CP, 20 without CP	Custom IMU (Inertial Measurement Unit)	• Sensor: MPU-9250 (Accel, Gyro, Mag.) • Microcontroller: Custom Arduino Pro Mini • Wireless: nRF24L01 (2.4 GHz RF) • Data Rate: 100 Hz • Battery: 3.7 V, 90 mAh (~3 h)	Two sensors placed: 1. Back of the hand 2. Above the wrist	Raw accelerometer and gyroscope data (time-domain) converted to frequency-domain features (Fast Fourier Transform). Feature vector (1 × 270) included amplitude, phase shift, and peak frequency of the first 5 harmonics for each sensor and axis.	To classify movement features associated with cerebral palsy (CP) from raw IMU data during a “stop sign” wrist motion task, distinguishing between children with and without CP	9 algorithms tested, including: • Random Forest • C4.5 Decision Tree • SVM, k-NN, MLP, etc.	• ~15 y.o.: 87.75% (Random Forest) • ~3 y.o.: 89.39% (C4.5 Decision Tree)	Machine learning applied to raw IMU data can successfully classify CP-related movement features without complex joint angle calculations. Decision-tree-based algorithms (Random Forest, C4.5) were most accurate. The system shows potential for accurate active range-of-motion assessment.	Provides a digital solution for classifying movement disorders like CP; could be used to monitor therapy effectiveness and for continuous at-home monitoring of hand movement patterns in children with movement disabilities.
Kim et al. [14]	26 children (14 males, 12 females); age: 6–12 years old; all physically healthy	Film-type pressure sensor (Force Sensing Resistor—FSR) array	64 (8 × 8) sensors; Sensor mat dimension: not fully specified, but sensor distance: 30 mm (w) × 30 mm (l)	Chair seat	Body pressure distribution data converted into 25 × 28 pixel heat map images.	Classification of 7 sitting postures: (a) sitting straight, (b) lean forward, (c) lean left, (d) lean right, (e) lean backward, (f) sitting at front of chair, (g) sitting crossed-legged	Convolutional Neural Network (CNN)	Accuracy: 97.5% (average from tenfold cross-validation; min: 0.970, max: 0.981). Recall and Precision for all postures > 0.9	The CNN algorithm was significantly superior (97.5% accuracy) to ANN (82.9%) and MNN (88.7%) for classifying children’s sitting postures using only seat pressure sensors. This confirms the applicability of CNN-based algorithms for smart chairs to support correct posture in children.	Development of smart chairs for children to monitor sitting posture in real time, helping to prevent musculoskeletal disorders and promote the formation of correct postural habits during childhood.
Lee et al. [28]	24 healthy children (11 boys, 13 girls); age: 7–12 years (mean = 10.13, SD = 1.62); country: South Korea	Custom film-type pressure sensor mat	• 64 (8 × 8) FSRs • Size: 318 × 318 mm • Frequency: 10 Hz • Resolution: 12-bit • Data transmission: Bluetooth	Seat pan of an adjustable children’s chair	2D pressure distribution maps (8 × 8 grid). Data was interpolated to 16 × 16 and normalized before being used as input.	Classification of nine sitting postures: good, leaning forward, leaning left, right foot over left, leaning right, left foot over right, sitting at front edge, slouching, crossed legs	Convolutional Neural Network (CNN)	Exp. 1 (User-specific): 99.66% Exp. 2 (All users, identifiable): 99.40% Exp. 3 (Unfamiliar user—Leave-one-out): 77.35% Good vs. Poor posture discriminator (unfamiliar user): PPV = 0.59, NPV = 0.95	A CNN applied to seat pan pressure distribution data is highly effective for classifying children’s sitting postures, especially when user-specific data is available. Performance drops for unfamiliar users but remains viable for good/poor posture discrimination.	Enables the development of a non-invasive, real-time sitting posture monitoring and correction system for children in classrooms or at home, helping to prevent musculoskeletal disorders by promoting postural awareness.
Bertoncelli et al. [29]	102 teenagers with CP (60 inpatients, 42 outpatients; 60 males); mean age 16.5 ± 1.2 years (range 12–18 yrs); with cognitive impairment and severe motor disorders	Clinical and functional assessment data from medical records and standardized scales	Data collected between 2006 and 2021; variables included type of etiology, spasticity, dystonia, epilepsy, neuromuscular scoliosis, hip dysplasia, GMFCS, MACS, EDACS	Two specialized hospitals (Nice, France)	Independent variables: type of etiology (ET), sex (SE), dystonia (D), spasticity (SP), epilepsy (E), neuromuscular scoliosis (NS), hip dysplasia (H), MACS, GMFCS, EDACS. Dependent variable: type of trunk muscle tone (hypotonic, spastic, normal).	To identify factors associated with hypotonic or spastic truncal tone (TT) in adolescents with CP	Multiple Logistic Regression (TT-PredictMed model)	Average Accuracy: 82% Sensitivity: 71% Specificity: 90%	The TT-PredictMed model successfully identified specific clinical factors (e.g., hip dysplasia, etiology, motor function scores) associated with hypotonic and spastic truncal tone. The model’s performance aligns with recent MLe applications in clinical diagnostics.	Enables clinicians to identify adolescents with CP at risk for specific types of postural instability (hypotonic/spastic TT), allowing for earlier, more personalized rehabilitation targeting trunk control.
Sukhadia & Kamboj [30]	43 infants (15 healthy, 28 with spastic cerebral palsy)	Custom IMU sensors (9 units)	Tri-axial accelerometer, gyroscope, and magnetometer; samples per second not specified	Nine sensors: both forearms, both upper arms, both lower legs, both upper legs, and trunk	Joint angles from limbs and trunk (3D data from 9 tri-axial sensors); stride length; leg dimension.	To detect spastic cerebral palsy (CP) based on posture and movement analysis	Support Vector Machine (SVM)	Accuracy: 88% (with 2-fold cross-validation)	An IMU-based system combined with machine learning can accurately identify infants with spastic CP by analyzing joint angles and movement parameters.	Enables early, automated detection of spastic CP using wearable sensors, facilitating timely intervention; serves as an objective tool to assist clinical diagnosis.
Li et al. [31]	Simulation study using a validated 6-year-old child occupant model (TUST IBMs 6YO-O); no human subjects	Finite Element (FE) vehicle crash simulation model; Machine Learning models	- FE Model: TUST IBMs 6YO-O (Hexahedron elements, detailed brain anatomy). - Simulation: LS-DYNA or similar explicit dynamics solver. - MLe Inputs: Collision speed (50–80 km/h), Sitting angle (90–135°). - Outputs: Head injury criteria (HIC15, 3 ms acceleration, BrIC, von Mises stress, Maxshear stress, MPS).	Virtual crash test environment (FRB—Frontal Rigid Barrier)	Input variables: impact speed, occupant sitting posture. Output (posture/injury) variables: head linear and rotational injury metrics (3ms accel., HIC15, BrIC), brain tissue stress/strain (von Mises, Maxshear, MPS).	To predict head injury risk and biomechanical response of a 6-year-old child occupant based on collision speed and sitting posture	LSTM, SVM, Random Forest (RF)	R^2^ > 0.93 for all models (LSTM, SVM, RF) on head injury indices (e.g., 3 ms accel., HIC15) using 10-fold cross-validation	The combination of simulation and machine learning provides a reliable predictive model for child head injury. The risk and primary mechanism of injury (linear vs. rotational load) are significantly influenced by both collision speed and sitting posture.	Enables virtual safety testing and optimization of child restraint systems (CRS) in autonomous vehicle scenarios by predicting injury outcomes for various postures and crash speeds, informing safer CRS design.
Franchak et al. [32]	15 infants (6–18 months, M = 11.28 months); 7 male, 8 female; laboratory study	MetaMotionR IMUs (Mbientlab); Later: Biostamp IMUs (MC10) in leggings (home case study)	3–4 IMUs; Accelerometer and Gyroscope; 50 Hz (Lab), 62.5 Hz (Home)	Right hip, thigh, and ankle (Lab); both hips and ankles (Home, embedded in leggings)	204 features from 4 s windows: 10 summary stats (mean, SD, skew, etc.) per sensor location, signal (accel/gyro), and axis; cross-sensor/axis correlations and differences.	Classify body position into 5 categories: supine, prone, sitting, upright, held by caregiver	Random Forest	Individual Models: 97.9% (Lab), ~86% (Home) Group Models: 93.2% (Lab)	Method accurately classifies infant body position and captures individual differences in time spent in each position; feasible for contactless, full-day home assessment.	Enables unobtrusive, long-form measurement of naturalistic infant motor behavior and posture in home settings, useful for developmental studies linking motor experience to other domains (e.g., language).
Eken et al. [33]	10 children (5 with HIV encephalopathy (HIVE), 5 typically developing (TD)); all girls; aged 5–12 years; GMFCS level II (HIVE group)	Video cameras (Bonita)	50 Hz; sagittal and frontal plane recordings; DeepLabCut (pre-trained ResNet101 model) for 2D markerless pose estimation	Laboratory setting; anatomical landmarks (shoulder, elbow, wrist, hip, chin) tracked in sagittal and frontal planes	Joint angles calculated from tracked landmarks: shoulder flexion/extension, elbow flexion/extension, shoulder abduction/adduction, trunk lateral sway. Joint angle trajectories and range of motion (ROM) over the gait cycle.	To classify and quantify differences in upper body postures and movements (arm swing, trunk sway) during gait between children with and without HIVE	Statistical Parametric Mapping (SPM); Mann–Whitney U test for ROM	SPM identified significant differences in joint angle trajectories: shoulder sagittal (20–59% gait cycle), shoulder frontal (80–93%), trunk frontal (44–65%). ROM significantly larger in HIVE group for shoulder abduction (*p* = 0.028) and trunk sway (*p* = 0.009).	Markerless tracking with DeepLabCut is feasible and sensitive for quantifying pathological upper body postures and movements during gait in children with HIVE, showing increased trunk sway and altered arm swing similar to other neurological disorders.	Serves as a low-cost, accessible alternative to conventional gait analysis for assessing postural deviations in clinical settings, especially in low-to-middle-income countries; useful for monitoring disease progression and therapy effectiveness.
Tao et al. [34]	514 primary school children (aged 6–12 years); 300 with forward head posture (FHP), 214 without; from 12 public schools in Nanjing, China	Kinect depth camera (Version: Kinect 2 for Windows); InBody 370 body composition analyzer; structured questionnaire.	Kinect: accuracy 0.001 m, standing distance 2 m; InBody: standard BIA protocol; Questionnaire: Chinese PAQ-A scale	Clinical/school setting; full-body frontal and lateral views	Anthropometric: age, sex, height, body weight, BMI. Lifestyle: daily and weekly homework time. Postural: Cervical Vertebrae Angle (CVA), ear-to-shoulder distance, horizontal angle of shoulders.	To predict the risk of forward head posture (FHP) disorder	Six algorithms tested: KNN, LGBM, XGBoost, RF, LM, SVM	RF AUC = 0.865 (best performer; LM AUC = 0.640 for comparison)	The Random Forest model demonstrated superior predictive accuracy for FHP. BMI, body weight, and age were the most influential predictors, with BMI being the most important.	Provides a tool for early screening and risk assessment of FHP in school-aged children, enabling targeted interventions focusing on weight management and monitoring of sedentary behaviors (e.g., homework time).
Ledwon et al. [35]	51 healthy infants aged 6–16 weeks; full-term, Apgar score 10	Sony HDR-AS200V camera	920 × 1080 px, 60 fps	Home setting; camera 1 m above infant	Six pose-based features: HBA, NPD, TBA, TAF, BPDv, BPDh (reduced to TBA, TAF, HBA via feature selection).	Automated classification of postural asymmetry (symmetry, left, right) in infants	QDA (best performer)	92.03% accuracy; 93.26% sensitivity; AUC: 0.913	The method provides quantitative, objective assessment of postural asymmetry with high sensitivity and agreement with expert judgment.	Screening tool for infant postural asymmetry; supports early neurodevelopmental assessment and therapy monitoring without additional tools.
Airaksinen et al. [36]	22 infants (mean age 6.7 months); typically developing; recruited for movement analysis	“Smart jumpsuit” with 4 wearable sensors (Movesense)	Inertial Measurement Units (IMUs): accelerometer and gyroscope; 52 Hz sampling rate; wireless via Bluetooth	Proximally on upper arms and legs (4 limbs)	Raw accelerometer and gyroscope signals (24 channels); 2.3 s windows with 50% overlap; used to classify posture and movement.	To automatically classify infant posture (prone, supine, side L/R, crawl posture) and gross body movements	Convolutional Neural Network (CNN) with iterative annotation refinement (IAR)	Posture: 94.1–99.1% UAR (depending on frame set) Movement: 71.9–82.4% UAR	The smart jumpsuit and CNN classifier achieve human-equivalent accuracy in posture and movement classification, demonstrating feasibility for automated infant movement assessment.	Enables objective, quantitative tracking of infant motor development in clinical and potentially home settings for early detection of neurodevelopmental risks.
Gama et al. [37]	• Real Infants: 2 healthy infants (1f, 1m), 8–25 weeks old, 16 videos (1440 annotated images) • Synthetic Infants: MINI-RGBD dataset, 12 synthetic infants, 1000 images each	RGB video cameras	Frame-by-frame and video input processing	In-lab/synthetic environment (supine position)	• 2D coordinates of body keypoints (e.g., eyes, shoulders, wrists, hips, knees, ankles). • Derived metrics: Object Keypoint Similarity (OKS), Average Precision/Recall (AP/AR), Neck-MidHip error, percentage of missing data/redundant detections, processing speed (fps).	To compare the performance of seven 2D human pose estimation methods for automatically estimating infant body posture from video	Seven Deep Neural Networks: AlphaPose, DeepLabCut/DeeperCut, Detectron2, MediaPipe/BlazePose, HRNet (BU & TD), OpenPose, ViTPose	Best Performer (ViTPose): • OKS: 0.92 (Real), 0.87 (Synth) • AP (average precision): 88.5 (Real), 73.7 (Synth) • AR (average recall): 90.9 (Real), 79.1 (Synth) • Neck-MidHip Error: ~6.0% (Real)	State-of-the-art pose estimation methods (especially ViTPose and HRNet-TD) work well on infant pose estimation without additional training. Performance varies significantly between methods in accuracy, missing/redundant detections, and speed. AlphaPose was the fastest (27 fps). DeepLabCut and MediaPipe performed poorly.	Enables automatic, markerless quantification of infant posture and movement from ordinary videos (e.g., from a smartphone). This is a key enabling technology for large-scale “in the wild” movement analysis, early screening for neurodevelopmental disorders (e.g., via GMA), and studying typical motor development.
Franchak et al. [38]	22 infants (4–14 months); 10 female, 12 male; 34 testing sessions (14 from younger group 4–7 mo, 20 from older group 11–14 mo)	MC10 Biostamp IMUs (Inertial Measurement Units)	4 IMUs; accelerometer and gyroscope; 62.5 Hz	Embedded in custom leggings on both hips (thighs) and both ankles	436 features from 4 s windows: 10 summary stats (mean, SD, skew, kurtosis, percentiles, etc.) per sensor location, signal (accel/gyro), and axis; cross-sensor/axis correlations and differences.	Classify body position into 5 categories: supine, prone, sitting, upright, held by caregiver	Random Forest	Individual Models: ~97.9% (Proximal), ~86% (Distal) Group Models: ~93.2% (Proximal)	Method accurately classifies infant body position and captures individual differences in time spent in each position. Feasible for contactless, full-day home assessment.	Enables unobtrusive, long-form measurement of naturalistic infant motor behavior and posture in home settings, useful for developmental studies linking motor experience to other domains (e.g., language).
Ali & Mohamed [39]	Infants (2–5 months post-term) from MINI-RGBD and RVI-38 datasets	RGB video cameras (Sony DSC-RX100), pose estimation software (MediaPipe, OpenPose, MeTRAbs)	25 FPS, 640 × 480 to 1920 × 1080 resolution	Clinical and home settings (supine position)	Joint angles (shoulder, elbow, hip, knee), movement velocity, acceleration, anti-gravity movements, symmetry of movement, postural variability.	Early prediction of cerebral palsy (CP) based on posture and movement patterns	SVM, NN, DT, Extra-Tree, XGBoost	MINI-RGBD: 91.67% (NN) RVI-38: 97.37% (SVM)	Pose estimation with MLe classifiers effectively distinguishes CP from typical development by quantifying movement and postural features.	Automated, non-invasive early screening for CP using widely available video recordings; suitable for home or clinical use.
Duda-Goławska et al. [40]	104 infants; longitudinal study at 4, 6, 9, 12 months; 301 visits analyzed	Xsens MTw Awinda IMUs	Accelerometer, gyroscope, magnetometer; 60 Hz (resampled from occasional 40 Hz)	Trunk and legs (optimal configuration)	1920 features from 5 groups: Statistical, Frequency, Summary, Differences, Correlations; extracted from 2 s sliding windows with 1 s overlap.	Classify infant body position into 5 classes: supine, sitting, upright, prone, hands and knees	CatBoost Classifier	F1 Scores (trunk and legs): sitting (0.942), upright (0.819), supine (0.955), prone (0.924), hands and knees (0.617).	CatBoost outperformed Random Forest. Statistical features (especially from accelerometer) were most important, followed by difference features. Sensor placement on trunk and legs was optimal.	Enables automatic, accurate monitoring of infant posture during naturalistic play; useful for assessing motor development and potentially detecting delays in lab, clinical, or home settings.
Rachwani et al. [41]	21 infants (10 girls, 11 boys) aged 6–10 months (M = 8.2 months); sitting experience ranged from 5 days to 4.75 months; all typically developing, born without complications	Video camera (home recordings via Zoom)	Resolution not specified; frame rate not specified	Home environment	Behavioral (video coded): Success in touching/grasping toy, falls, hand support, changes in base of support. Kinematic (DeepLabCut): 2D coordinates of wrist, shoulder, hip; initial trunk angle, mean trunk angle, trunk angular displacement, reach time, normalized reach path, reach velocity, straightness score.	To quantify postural control (trunk kinematics) and its relation to successful multi-directional reaching during unsupported sitting	DeepLabCut (for pose estimation) and Custom MATLAB program (for kinematic variable calculation); statistical analysis (ANCOVA) performed in SPSS (version 28)	Behavioral: Touch: 100% (both directions) Grasp: ~94% (both directions) Falls: ~3% (both directions) Kinematics: No significant differences in trunk displacement or reaching kinematics between directions or across sitting experience	All infants, including novice sitters, were successful at reaching in both directions, demonstrating functional multi-directional postural control from the onset of independent sitting. Posture became more upright with experience, but arm and trunk movements during reaching were similar regardless of sitting skill level.	Provides an objective method (video-based pose estimation) to assess functional sitting postural control via multi-directional reaching. Suggests therapeutic strategies for sitting acquisition should involve variable practice in all planes of motion from early stages, rather than a specific sequence.

Notes. MLe: machine learning; DL: deep learning; MAIJU: Motor Assessment of Infants with a Jumpsuit; ASD: autism spectrum disorder; COP: center of pressure; AP: anteroposterior; ML: mediolateral; ANOVA: analysis of variance; AUC: area under the curve; ViTPose: Vision Transformer for Pose Estimation; FSR: force sensing resister; CNN: Convolutional Neural Network; NB: Naïve Bayes Classifier; DT: Decision Tree; NN: Neural Network; MLR: Multinomial Logistic Regression; SVM: Support Vector Machine: LSTM: Long Short-Term Memory; GRU: Gated recurrent unit; BiLSTM: bidirectional long short-term memory; BiGRU: bidirectional gated recurrent unit; CP: cerebral palsy; MIT: Minimising Impairment Trial; iWHOT: Infant Wrist Hand Orthosis Trial; k-NN: k-nearest neighbors; MLP: multilayer perceptron; MNN: multi-layer neural network; PPV: positive predictive value; NPV: negative predictive value; SD: standard deviation; GMFCS: Gross Motor Function Classification System; MACS: Manual Ability Classification System; EDACS: Eating and Drinking Ability Classification System for Individuals with Cerebral Palsy; IMU: Inertial Measurement Unit; BrIC: Brain Injury Criterion; MPS: Maximum Principal Strain; KNN: K-nearest neighbor; LGBM: light gradient boosting machine; XGBoost: extreme gradient boosting; LM: linear model; HBA: Head Bend Angle; NPD: Nose Point Distance; TBA: Trunk Bend Angle; TAF: Trunk Asymmetry Factor; BPDv: Bend Point Distance horizontal; BPDh: Bend Point Distance vertical; QDA: Quadratic Discriminant Analysis; UAR: unweighted average recall; BU: Bottom-Up; TD: Top-Down; GMA: General Movement Assessment; FPS: frames per second; ANCOVA: Analysis of Covariance.

**Table 4:** Comparative summary of machine learning models by task, sensing modality, dataset, and performance metrics.

Task/Application	Algorithm(s)	Sensing Modality	Dataset/Sample Size	Key Metrics (Accuracy/F1/AUC/Other)	Inference/Processing Time	Reference(s)
Sitting posture classification	CNN (LeNet-5), SVM, NB	Pressure mat (8 × 8 FSR)	10 children	Acc = 95.30% (CNN); SVM = 94.20%	~20 ms per frame (real time)	Kim et al. [12]
Sitting posture classification (7 classes)	CNN	Pressure mat (8 × 8 FSR)	26 children	Acc = 97.50%; Precision > 0.90 (all classes)	~15 ms per frame	Kim et al. [13]
Sitting posture classification (9 classes)	CNN	Pressure mat (8 × 8 FSR)	24 children	Acc = 99.66% (user-specific); 77.35% (unfamiliar)	Real-time feasible	Lee et al. [28]
ASD identification	Naïve Bayes	Force plate (COP features)	50 children (25 ASD, 25 TD)	Acc = 90.00%; Sens = 82.60%; Spec = 100%; F1 = 0.90	<1 s per trial	Li et al. [14]
Gross motor development (delay detection)	Random Forest	Video (home)	90 infants	Acc = 94.00%; F1 = 0.94; AUC = 0.98	~0.5 s per segment	Yang et al. [26]
Infant posture classification (prone, supine, side, crawl)	CNN	Wearable IMUs (smart jumpsuit)	22 infants	Acc = 94.10–99.10% (UAR)	Near real time	Airaksinen et al. [35]
Gross motor milestone detection	SVM + LME	Multi-sensor wearable (MAIJU)	134 infants	Acc = 90.90–96.80%; ρ(age, BIMS) = 0.93	Real time (mobile)	Airaksinen et al. [25]
CP detection (wrist motion)	RF, C4.5 DT	IMUs (hand/wrist)	140 children (89 + 51 CP)	Acc = 87.75–89.39%	~50 ms per sample	Khaksar et al. [15]
CP subtype (hemiplegia vs diplegia)	BiGRU	Force plate (COP series)	57 children (CP)	Acc = 76.43%	N/R	Arias Valdivia et al. [27]
CP detection (pose estimation)	SVM, NN	RGB video	MINI-RGBD and RVI-38 datasets	Acc = 91.67–97.37%	~25 fps (video)	Ali & Mohamed [36]
Forward head posture risk prediction	Random Forest	Depth camera + BIA	514 school children	AUC = 0.865; Acc ≈ 86%	<1 s per case	Tao et al. [33]
Postural asymmetry detection	QDA	Video (home)	51 infants	Acc = 92.03%; Sens = 93.26%; AUC = 0.913	~0.3 s per frame	Ledwoń et al. [34]
Infant posture classification (home IMUs)	Random Forest	Wearable IMUs (leggings)	15–22 infants	Acc = 86.00–97.90% (individual models)	Real time	Franchak et al. [16,17]
Infant pose estimation benchmark	ViTPose, HRNet, AlphaPose, etc.	RGB video (2D pose estimation)	MINI-RGBD + real infant videos	OKS = 0.92; AP = 88.50; AR = 90.90 (Real)	27 fps (best model)	Gama et al. [19]
Head-injury risk prediction (sitting posture/impact)	LSTM, SVM, RF	Finite-element simulation model	Synthetic (6-year-old model)	R^2^ > 0.93 (all indices)	N/R	Li et al. [31]

Notes. Acc: accuracy; AUC: area under the ROC curve; F1: F1-score; OKS: Object Keypoint Similarity; ρ: Spearman’s correlation; Sens: sensitivity; Spec: specificity; N/R: not reported; CNN: Convolutional Neural Network; SVM: Support Vector Machine; RF: Random Forest; DT: Decision Tree; QDA: Quadratic Discriminant Analysis; IMU: inertial measurement unit; BIA: bioelectrical impedance analysis; UAR: unweighted average recall; BIMS: BABA infant motor score, BiGRU: Bidirectional Gated Recurrent Unit.

## Data Availability

No new data were created or analyzed in this study.
